# Rapid Multi-Residue Detection Methods for Pesticides and Veterinary Drugs

**DOI:** 10.3390/molecules25163590

**Published:** 2020-08-07

**Authors:** Min Jia, Zhongbo E, Fei Zhai, Xin Bing

**Affiliations:** 1 Key Laboratory of Animal Resistance Biology of Shandong Province, Institute of Biomedical Sciences, Key Laboratory of Food Nutrition and Safety of Shandong Normal University, College of Life Science, Shandong Normal University, Jinan 250014, China; ezb1234@126.com (Z.E.); zhaifeijay@163.com (F.Z.); 2 Shandong Product Quality Inspection Research Institute, Jinan 250012, China; bingxinhaida@163.com

**Keywords:** multi-residue detection, pesticide and veterinary drug residues, recognition element, inherent characteristic

## Abstract

The excessive use or abuse of pesticides and veterinary drugs leads to residues in food, which can threaten human health. Therefore, there is an extremely urgent need for multi-analyte analysis techniques for the detection of pesticide and veterinary drug residues, which can be applied as screening techniques for food safety monitoring and detection. Recent developments related to rapid multi-residue detection methods for pesticide and veterinary drug residues are reviewed herein. Methods based on different recognition elements or the inherent characteristics of pesticides and veterinary drugs are described in detail. The preparation and application of three broadly specific recognition elements—antibodies, aptamers, and molecular imprinted polymers—are summarized. Furthermore, enzymatic inhibition-based sensors, near-infrared spectroscopy, and SERS spectroscopy based on the inherent characteristics are also discussed. The aim of this review is to provide a useful reference for the further development of rapid multi-analyte analysis of pesticide and veterinary drug residues.

## 1. Introduction

Pesticides and veterinary drugs are indispensable for increasing food production, as well as improving animal breeding and aquaculture [1]. Pesticides are widely applied in modern agriculture to control weeds and pests and regulate the growth of plants [2]. Veterinary drugs are commonly administered in farming practices for the prevention and treatment of diseases and promoting growth [3]. The application of pesticides and veterinary drugs can prevent devastating losses in agriculture and animal husbandry industries, enabling them to meet the demands of a rising global population [4,5]. However, the excessive use or abuse of pesticides and veterinary drugs can lead to drug residues in food and the environment, which can threaten human health through food chains [6,7]. Therefore, effective detection methods for residues have been developed for monitoring food safety and ensuring public health.

The traditional methods for pesticide and veterinary drug residues determination are usually based on instrumental techniques such as gas chromatography (GC), high performance liquid chromatography (HPLC), or chromatographic methods coupled with mass spectrometry (MS) detectors [8]. These methods provide abundant qualitative and quantitative information of the residues with high accuracy. However, these systems are limited by complicated sample pre-treatments and require highly trained technicians and expensive equipment [8]. On the other hand, rapid methods such as immunoassays [9], spectroscopic analyses [10], and electrochemical techniques [11] provide relatively convenient and highly sensitive strategies for the determination of pesticides and veterinary drugs. Although the accuracy and precision of rapid methods are not as good as those of instrumental techniques, these methods can be used complementarily to instrumental methods, particularly as pre-screening methods for detection in large-scale samples. Therefore, novel analysis methods for the rapid and sensitive detection of pesticides and veterinary drugs are highly desired.

Common rapid methods are capable of detecting a single target with high specificity [12], whereas real food samples generally always contain more than one pesticide or veterinary drug. Therefore, multi-residue detection methods are more favorable for the actual analytical needs of end-users. Meanwhile, these simultaneous detection strategies are well-suited for screening analyses with the characteristics of ease of use, high-throughput, and low cost per sample [13].

**Figure 1:**
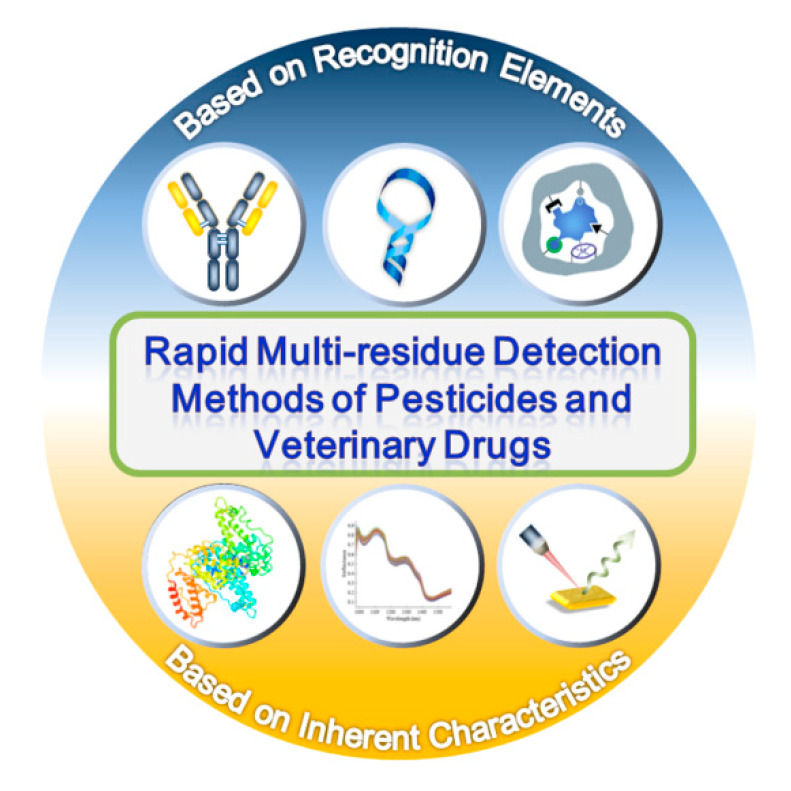
Rapid multi-residue detection methods of pesticides and veterinary drugs.

In this review, two strategies of rapid multi-residue methods are addressed for the measurement of pesticides and veterinary drugs (Figure 1). The first strategy is rapid multi-residue methods based on different recognition elements. The preparation and application of antibodies, aptamers, and molecular imprinted polymers (MIPs) with broad specificity are summarized. The second strategy consists of rapid multi-residue detection methods based on the inherent characteristics of pesticides and veterinary drugs. In this category, enzymatic inhibition-based sensors, near-infrared (NIR) spectroscopy, and SERS spectroscopy are introduced.

## 2. Rapid Multi-Residue Detection Methods Based on Different Recognition Elements

The accurate determination of trace target analytes in complex food matrices is a major challenge for the development of rapid detection methods [14]. The use of recognition elements can overcome this challenge, providing the characteristics of high affinity and specificity to target analytes. Therefore, the recognition elements are primarily responsible for the performance of the methods. In recent studies, the most popular affinity-based recognition elements include antibodies, aptamers, and MIPs. To achieve multi-residue detection, the recognition elements should have broad specificity with respect to different individual targets. Thus, broadly specific recognition elements with the property of recognizing various analytes have been developed.

### 2.1. Rapid Multi-Residue Detection Methods Based on Antibodies

**Figure 2:**
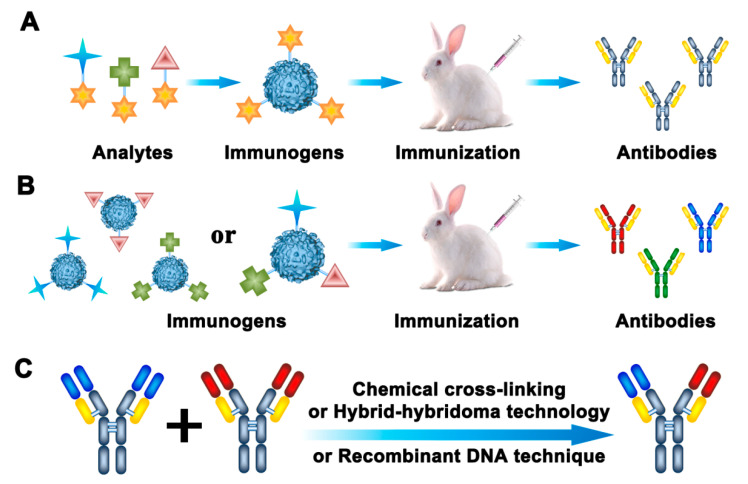
Generation strategies for broad specific antibodies. (**A**) Generic antibodies; (**B**) broad-spectrum antibodies; (**C**) bispecific antibodies.

Antibodies have long been the most common recognition elements used in rapid detection methods. Antibody-based approaches, such as the enzyme-linked immunosorbent assay (ELISA), have been utilized as alternatives to the routine techniques for the detection of pesticide and veterinary drug residues in food and environmental samples. For multi-residue detection, there are four ways to obtain broadly specific antibodies. Generic antibodies prepared by “general-structure” immunogens are one kind of broadly specific antibodies (Figure 2A). Broad-spectrum antibodies generated with multi-hapten immunogens are another kind of broadly specific antibodies (Figure 2B). With the development of genetic manipulation and hybridoma technology, bispecific antibodies composed of two different heavy/light chains have been widely used as broadly specific antibodies (Figure 2C). The last way to obtain broadly specific antibodies is by combining numerous different analyte-specific antibodies together, in order to recognize different individual targets.

#### 2.1.1. Preparation and Application of Generic Antibodies

Generic antibodies are generated by immunization with “general-structure” haptens, which exhibit the common features of an entire class of related analytes [15], as shown in Figure 2A. Therefore, generic antibodies have a broad cross-reactive pattern for structurally similar analytes. The design of a suitable hapten is a critical step for creating a generic antibody and developing a broadly specific immunoassay. Generic haptens should preserve the original steric and electronic properties of all or most of the target analytes [16]. However, various haptens can be designed for the same group of analytes. Thus, the obtained antibodies could show different antibody characteristics and recognition abilities; some of these antibodies may not even show the desired broad specificity.

To solve this problem, several strategies have been developed to obtain the desired antibodies [13]. Taking organophosphorus pesticides (OPs) for example, the commonly used OPs usually contain an aromatic ring and a thiophosphate moiety. Therefore, generic haptens containing an *O*,*O*-diethyl thiophosphate moiety and a benzene ring were designed by two separate research groups [17,18,19]. Satisfactory broad specificity and significantly improved sensitivity were obtained in the ELISA methods. Furthermore, Zhao et al. [20] synthesized eight kinds of *O*,*O*-diethyl *O*-(3-carboxyphenyl) phosphorothioate with different carboxy groups in the meta position of the benzene ring, in order to prepare immunogens or coating antigens. A broadly specific monoclonal antibody was generated, which showed high and uniform sensitivity to seven *O*,*O*-diethyl OPs and six *O*,*O*-dimethyl OPs. These trial-and-error based hapten design methods are often unpredictable, time-consuming, and expensive. This is because of the required simple chemical structure comparisons at the two-dimensional (2D) level when the haptens are designed. With the development of computer-assisted molecular modeling, the influences of the hapten structures and spacers on steric and electronic properties can be investigated and evaluated at the three-dimensional (3D) level [21]. The potential binding sites and modes of antibody-analyte recognition can be predicted by molecular docking and computational chemistry techniques [22]. With the help of these novel computer-assisted strategies, the most appropriate hapten chemical structure can be selected without a large number of experiments [23]. Xu et al. [24] used 2D and 3D quantitative structure-activity relationships (QSARs) to increase the sensitivity and investigate antibody recognition. By applying molecular modeling, two heterologous haptens for Ops were designed and synthesized. A broadly specific antibody was designed and used to develop an ELISA method, which demonstrated excellent sensitivity for eight *O*,*O*-diethyl Ops. Molecular modeling can also assist immunochemists design the most appropriate hapten for other pesticides and veterinary drugs. The generic antibodies of sulfonamides (SAs) and fluoroquinolones (FQs) with a wide recognition profile have also been developed using molecular modeling and theoretical tools.

Various immunochemical methods can be applied for the detection of pesticide and veterinary drugs. ELISA involving antibodies combined with enzymatic markers has taken a leading position in immunoassay methods [25]. Due to the low molecular weight of the target analytes, competitive ELISA is commonly used for pesticide and veterinary drug residue determination [26,27]. A monoclonal antibody for five antibacterial synergists, 5C4, with uniform broad specificity and high affinity was obtained and used to develop an indirect competitive ELISA (icELISA) method [28]. The IC_50_ values were 0.067–0.139 μg/L, with cross-reactivity of 48.2–418.7% for the five antibacterial synergists under optimal conditions. Due to the icELISA requiring enzyme-labeled secondary antibodies and complicated procedures, the 5C4 antibody was also used to develop a direct competitive ELISA (dcELISA) method [29]. The IC_50_ values were 0.208–9.24 μg/L, that is, 1.36–31.16 fold higher than those of icELISA. However, the dcELISA method required lower testing costs and less time for the detection of multiple antibacterial synergists in real samples. For improvement of the sensitivity of ELISA, chemiluminescence enzyme immunoassays have been developed with luminol as a chemiluminescence substrate. Zeng et al. [30] utilized pazufloxacin as a FQs generic hapten to develop a broadly specific antibody with broad cross-reactivity ranging from 5.19% to 478.77% with 21 kinds of FQs. An indirect competitive chemiluminescence enzyme immunoassay was developed with a limit of detection (LOD) of 0.10–33.83 ng/mL and recovery of 84.6–106.9% in milk samples, which exhibited high sensitivity and suitability for screening FQs in real samples.

In addition to ELISA methods, lateral flow immunoassay (LFIA) is another conventional immunochemical method [31,32], which has been widely used for the on-site detection of residues. LFIA offers several advantages, including ease of use, low time consumption, convenience, low cost, and high-throughput screening for several target analytes [33]. The most commonly used LFIA method employs gold nanoparticles (AuNPs) as reporters for colorimetric detection. A LFIA method using a broadly specific anti-adamantanes monoclonal antibody has been developed for five adamantanes, with visual LODs of 0.1–10 μg/kg (similar to the results of ELISA) [34]. To further improve the sensitivity of LFIA, other labels such as fluorescent nanoparticles have been employed in LFIA fabrication. An ultrasensitive fluorescent LFIA has been prepared with a nonspecific monoclonal antibody [35]. This fluorescent LFIA can rapidly screen for seven β-agonists within 8 min with an LOD lower than 50 pg/g of pork.

Generic antibodies have successfully been used in multi-analyte assays for pesticide and veterinary drug residues. However, the desired generic immunogen used to produce generic antibodies with broad specificity and high affinity are difficult to design. Meanwhile, the generic antibody cannot have a consistent affinity for all of the analytes, which leads to different relative responses to the target analytes in the detection methods. In addition, the developed assays cannot distinguish each analyte respectively when the analytes are mixed, as is typically the case in real samples. Furthermore, generic antibodies can be produced by the same class of analytes with a general structure. Thus, broad specificity antibodies against different classes of target analytes cannot be obtained using this approach.

#### 2.1.2. Preparation and Application of Broad-Spectrum Antibodies

In order to broaden the recognition spectrum of antibodies, multi-hapten strategies have been used to produce various antibodies in one experimental animal. By using this approach, broadly specific antibodies against different classes of pesticide and veterinary drug residues can be produced. A multi-hapten immunogen can be obtained by two approaches: (1) Several different haptens are coupled to one carrier protein simultaneously [36]; or (2) each hapten is coupled to a carrier protein individually and, then, the multiple immunogens are used for simultaneous immunization [37] (Figure 2B).

By using the first approach, four multi-hapten antigens for parathion have been conjugated to bovine serum albumin (BSA) to generate a broadly specific antibody. The sensitivity of ELISA decreased, due to the increase of structural complexity. However, the specificity of other target analytes was broadened in this immunoassay [38]. Furthermore, to simultaneously screen two classes of Ops residues, a dual-generic hapten immunogen was generated by coupling two generic haptens (*O*,*O*-dimethyl thiophosphate hapten and *O*,*O*-diethyl phosphate hapten) to BSA. A broadly specific polyclonal antibody showed selectivity to eleven organothiophosphate pesticides and five organophosphate pesticides [39]. Using the second approach, three 2-(2-aminoethyl) benzimidazole, 2-benzimidazole propionic acid, and 2-mercaptobenzimidazole have been conjugated to keyhole limpet hemocyanin, and the resulting mixture was used as the immunogen [40]. The obtained antibody could recognize carbendazim and other benzimidazole-type fungicides. To improve the sensitivity of the antibody, a hybridoma screening method was used to develop monoclonal antibodies against six FQs which could recognize both individual and multiple analytes [41].

Broad-spectrum antibodies have also been used to develop immunoassays for high-throughput screening of pesticide and veterinary drug residues. An icELISA method was developed with polyclonal antibodies against dichlorvos and paraoxon [42]. The LODs and recovery values in real samples were determined with desirable results. Peng et al. [43] developed an LFIA for the simultaneous detection of 32 FQs using a broadly specific monoclonal antibody prepared from a mixture of a norfloxacin immunogen and a sarfloxacin immunogen. All 32 FQs analytes could be detected within 10 min with a visual LOD ranging from 0.1 to 10 ng/mL.

Compared with generic antibodies, broad-spectrum antibodies have broader recognition spectra and do not require the design of a generic antigen. However, due to antigenic competition and the low coupling ratio of each antigen in multi-hapten strategies, lower affinities to analytes are obtained than that in antibodies derived from a single immunogen. The higher total number of hapten molecules on the surface of a multi-hapten immunogen may not be advantageous for lymph cells to recognize the carrier protein, which would result in poor induction of the immune system [44]. Meanwhile, it is technically difficult and uncontrollable to obtain optimal immunogens with equal hapten availability in animal immune systems.

#### 2.1.3. Preparation and Application of Bispecific Antibodies

Bispecific antibodies contain two different antigen-binding sites, which can detect two distinct residues [45] (Figure 2C). Bispecific antibodies can be generated by chemical cross-linking, hybrid-hybridoma technology, or recombinant DNA techniques [46]. Ouyang et al. [47] applied a hybrid-hybridoma strategy to produce a bispecific monoclonal antibody, which could bind two kinds of pesticides—methyl parathion and imidacloprid—simultaneously. This bispecific antibody showed good affinity and specificity to the target analytes. To further broaden the recognition spectrum, bispecific monoclonal antibodies associated with antigen-binding sites for two classes of pesticides were developed to detect eight OPs pesticides and two neonicotinoid insecticides [48]. Furthermore, a recombinant DNA technique has been used to fuse two parent recombinant single-chain variable fragments which recognize FQs and SAs to form a recombinant bispecific antibody [49]. The antibody retained the affinity features of the parent antibodies, which allowed for the simultaneous analysis of 20 FQs and 14 SAs. Based on phage display technology, the recombinant antibodies have been selected from antibody libraries directly without selection of hybridoma cell lines. The diversity of phage antibody libraries from hybridoma cell lines should be very large to increase the possibility of selecting a desirable antibody [50]. A recombinant antibody variable fragment with broad specificity for OP pesticides, which was obtained by cloning VL and VH genes from hybridoma cells secreting monoclonal antibody, was used to develop a one-step ELISA for the detection of parathion, phoxim, quinalphos, and dichlofenthion [51].

Several multi-analyte immunoassays for the screening of pesticide and veterinary drug residues have been developed using bispecific antibodies. A bispecific antibody-based icELISA method was developed for the detection of 5-morpholinomethyl-3-amino-2-oxazolidone and leucomalachite green in aquatic products [52]. The LODs for 5-morpholinomethyl-3-amino-2-oxazolidone and leucomalachite green were 0.2 and 4.8 ng/mL, respectively. In addition to the conventional icELISA, a chemiluminescent ELISA method using a bispecific monoclonal antibody was developed for analysis of methyl parathion and imidacloprid [47]. The linear ranges for the two target analytes were both 1.0–500 ng/mL, with an LOD of 0.33 ng/mL.

Bispecific antibodies can realize the simultaneous analysis of two different classes of pesticide and veterinary drug residues. However, bispecific antibodies are difficult to obtain. Due to the co-dominant expression of immunoglobulin genes, ten different antibody species were obtained with only one contributing to the desired bispecificity. This drawback led to the production of a large percentage of non-functional antibodies [53]. Therefore, elaborate purification steps are required to obtain the desired bispecific antibody. Moreover, compared with natural monoclonal antibodies, bispecific antibodies have disadvantages in terms of the sensitivity and stability.

#### 2.1.4. Preparation and Application of Multi-Antibodies

In addition to generating the above three kinds of broadly specific antibodies, an easy way to realize the multi-analyte analysis of residues is to incorporate numerous specific antibodies into a single test. Individual antibodies or antigens could be distinguished by position or by labeling with different labels. Through the flexible combination of antibodies, analytes with very different structures can be detected simultaneously. Moreover, generic antibodies, broad-spectrum antibodies, and bispecific antibodies can also be utilized in combination to develop multi-analyte analyses, which can further broaden the recognition spectrum of antibodies. In contrast to multi-analyte assays using the above three kinds of broadly specific antibodies, assays based on multi-antibodies can distinguish each analyte semi-quantitatively or quantitatively. Single-label and multi-label strategies have been applied in multi-analyte assays.

**Figure 3:**
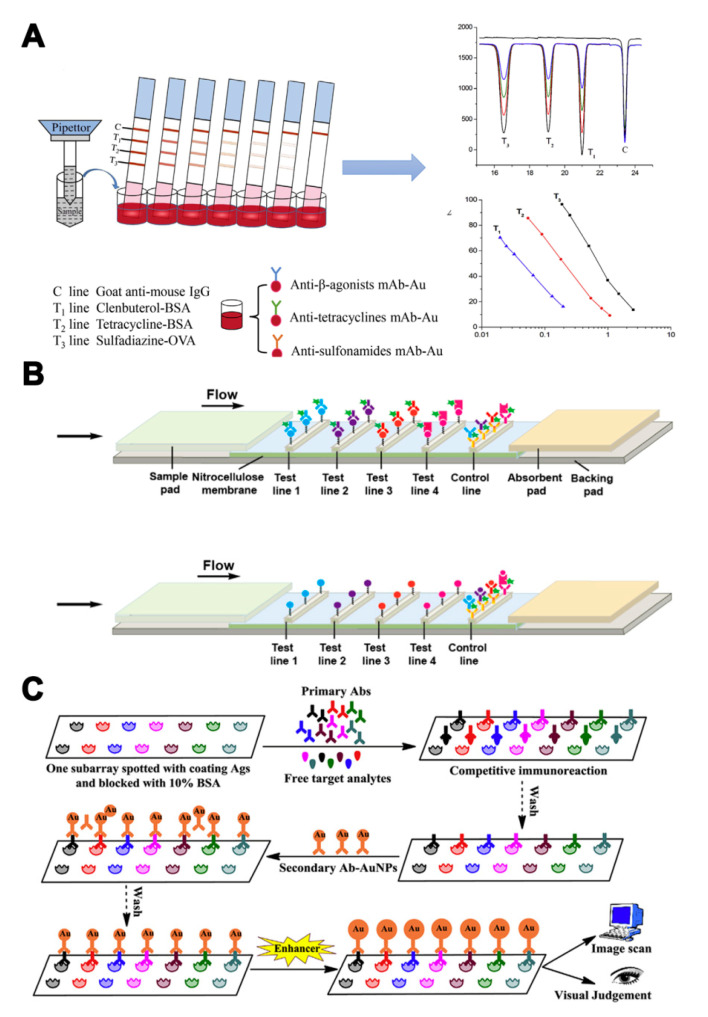
The assays based on multi-antibodies in single-label strategies. (**A**) Illustration of colorimetric assays for multi-residues detection using a AuNPs-based multiplex lateral flow immunoassay (LFIA) [54]. (**B**) Illustration of a multiplex near-infrared (NIR) fluorescence-based LFA for the detection of four families of antibiotics [55]. (**C**) Illustration of a competitive immunochip assay based on Nanogold-labeled antibodies for simultaneous detection of seven pesticides [57]. Reproduced with permission from [54]. Copyright Elsevier, 2016. Reproduced with permission from [55]. Copyright Elsevier, 2016. Reproduced with permission from [57]. Copyright Elsevier, 2016.

As different antibodies can be coated on different zones of biochips or lateral flow strips, single-label strategies can be carried out in LFIA or biochip microarray assays to realize multi-analyte analysis. Han et al. [54] developed a multiplex LFIA for the simultaneous determination of β-agonists, SAs, and tetracyclines (TCs). Three test lines with three antigens were located on the strip membrane. AuNPs labeled antibodies were mixed in microwells (Figure 3A). The sample could be directly detected, without pre-treatment, within 10 min. Combined with portable photometric strip readers, quantitative determination of target veterinary drug residues is possible. Aside from visual detection, other detection modes have also been used in multi-analyte analyses by changing the conjugated labels, such as NIR labels and Raman active labels. A NIR fluorescence-based multiplex LFIA was developed to detect four families of antibiotics (β-lactams, TCs, quinolones, and SAs) [55]. NIR fluorescence was used to label broad-specificity monoclonal antibodies. Different antigens were immobilized on different zones of a nitrocellulose membrane to serve as capture reagents (Figure 3B). The cut-off values of the four families of antibiotics were in the range of 2–8 ng/mL, much lower than that in conventional AuNPs-based LFIA. Moreover, Shi et al. [56] applied AuNPs conjugated to 4-aminothiophenol as a surface-enhanced Raman scattering (SERS) label to develop a SERS-based multiple LFIA for the simultaneous detection of neomycin and quinolones antibiotics. The sensitivity of this method was further improved, with LODs down to 0.37 and 0.55 pg/mL, using SERS. To further increase the number of target analytes, microarray biochips have become a promising tool for monitoring pesticide and veterinary drug residues. A microarray chip has been developed for the simultaneous detection of seven pesticides (triazophos, methyl-parathion, fenpropathrin, carbofuran, thiacloprid, chlorothalonil, and carbendazim) [57]. Seven antigens were dispensed onto separate test zones of a nitrocellulose membrane. Nanogold was applied for signal amplification, which could be evaluated by naked-eye assessment and image analysis (Figure 3C). The visual LODs ranged from 1–100 ng/mL and LODs for the pesticides were 0.02–6.45 ng/mL. In addition to LFIA or biochip microarray assays, a hybrid assay based on two broadly specific monoclonal antibodies with different spectra of SAs recognition was engineered in a sandwich configuration using antibody−hapten conjugates. The monoclonal antibodies were labeled by a horseradish peroxidase. Due to the summation effect in formation of a two- monoclonal antibodies sandwich complex, simultaneous determination of 14 SAs was achieved in turkey muscle and milk samples [58].

**Figure 4:**
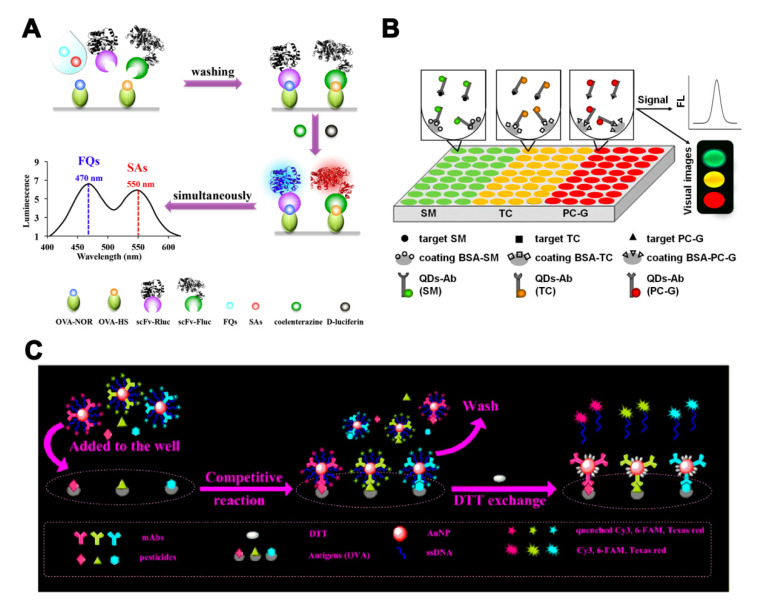
The assays based on multi-antibodies in multi-label strategies. (**A**) Illustration of a bioluminescent ELISA based on dual-luciferases for the detection of fluoroquinolones (FQs) and sulfonamides (SAs) [59]. (**B**) Illustration of a multicolor quantum dot (QD)-based microtiter plate array analysis for three kinds of antibiotics [60]. (**C**) Illustration of a multi-analyte FLISA based on oligonucleotide signal amplification for simultaneous detection of three small-molecule contaminants [61]. Reproduced with permission from [59]. Copyright Elsevier, 2018. Reproduced with permission from [60]. Copyright Elsevier, 2015. Reproduced with permission from [61]. Copyright Elsevier, 2020.

Multi-label strategies have a wider range of applications, compared with single-label strategies. In addition to LFIA and biochip microarray assays, ELISA and fluorescence-linked immunosorbent assay (FLISA) can also realize quantitative analysis for each analyte by using a multi-label strategy. Yu et al. [59] developed a dual-luciferases bioluminescent ELISA for simultaneous screening of the 20 FQs and 21 SAs residues with only one substrate addition step. Two luciferases—firefly luciferase and Renilla luciferase—were used to label broadly specific single-chain variable fragments against FQs and SAs (Figure 4A). This method enabled multiplex detection of pesticide residues with a simple operation sequence and high throughput. Quantum dots (QDs) and fluorophores with different emission wavelengths can also be used in multi-label strategies. A multicolor QD-based microtiter plate array analysis has been used for the sensitive and visual detection of streptomycin, TC, and penicillin G in milk [60]. Three QDs with different emission wavelengths were conjugated with antibodies for analytes recognition and signal amplification (Figure 4B). The sensitivity of analysis was improved and the LOD for each antibiotic was 5 pg/mL. Zhang et al. [61] employed fluorescently labeled oligonucleotide and an AuNP signal amplification strategy to develop a multi-analyte FLISA for the detection of three OPs (triazophos, parathion, and chlorpyrifos). Three antibodies and their corresponding fluorophore-labeled oligonucleotides were conjugated on AuNP probes. The coating antigens of three OPs in microtiter plate wells competed with the OPs in the sample to bind the antibodies on AuNP probes. The oligonucleotides were released after the addition of DTT and recovered the quenched fluorescence (Figure 4C).

Multi-analyte analysis based on multi-antibodies can realize high-throughput, highly efficient, rapid, and quantitative analyses. However, multiplex antibodies and different labels result in an inevitable increase of detection cost. Meanwhile, it is not easy to obtain good sensitivity to different targets in multi-analyte assays. The ideal ratio of mixed antibodies should be carefully optimized. Moreover, cross-reactions with other analogues should be avoided when using mixed antibodies.

### 2.2. Rapid Multi-Residue Detection Methods Based on Aptamers

Aptamers serve as an ideal alternative to an antibody for the analysis of pesticide and veterinary drug residues [62]. Aptamers are single-stranded oligonucleotides (RNA or DNA) selected by the Systematic Evolution of Ligands by the Exponential enrichment (SELEX) method [63]. Aptamers can fold into a special 3D conformation which can non-covalently bind to target molecules with high affinity and specificity [64]. Aptamers have several advantages over antibodies, such as chemical stability, ease of synthesis and modification, low molecular weight, and wider target spectra [11]. Therefore, aptamers have been widely used as recognition elements for developing biosensors in food safety monitoring and detection. With the development of aptamer screening and optimization techniques, several broad specific aptamers have been selected and designed. Broadly specific aptamers can be divided into four kinds by different production strategies: (1) Group-specific aptamers selected through the common chemical structure of a class of molecules; (2) broad-spectrum aptamers selected through multiplex targets; (3) truncated aptamers with broad specificity obtained by aptamer truncation; and (4) multi-aptamers by combined application of different aptamers.

#### 2.2.1. Preparation and Application of Group-Specific Aptamers

Aptamers usually have high specificity for target molecules which allow them to only be used for detecting specific targets. As it is impossible to select aptamers for all of the analytes, being able to generate aptamers that can recognize a group of molecules with a common structure would be significant for the detection of residues [65]. By rational design of the initial targets, group-specific aptamers can be selected with general specificity for a group of analytes [66]. Nikolaus et al. [67] used kanamycin (KAN) A as a target molecule in the Capture-SELEX procedure. KAN A-specific aptamers and group-specific aptamers binding to various aminoglycoside antibiotics were derived from the SELEX procedure. The aptamer #11_76, with the broadest spectrum, could bind to six kinds of aminoglycoside antibiotics. Fluorescence aptamers have been used to develop microplate-based assays to detect aminoglycoside antibiotics in real waste water samples.

#### 2.2.2. Preparation and Application of Broad-Spectrum Aptamers

Broad-spectrum aptamers can be derived from conventional SELEX procedures, with a little modification, by changing the single target to multiplex targets [68]. By using this strategy, many broad spectrum aptamers for pesticides and veterinary drugs have been selected. A broad-spectrum aptamer against TC, oxytetracycline, and doxycycline was selected by using TC and doxycycline as targets [69]. Then, an aptamer that could bind to iprobenfos and edifenphos was selected from the modified immobilization-free GO-SELEX, using these two analytes as targets [70]. Furthermore, aptamers SS2-55 and SS4-54, demonstrating high affinities and specificities to the four OP pesticides, were isolated by a SELEX procedure using phorate, profenofos, isocarbophos, and omethoate as targets [65].

Various aptasensors have been developed to screen pesticides and veterinary drugs. For example, the above OP broad-spectrum aptamer SS2-55 has been used in colorimetric, electrochemical, and SERS assays [65]. AuNPs colorimetric assay combined with aptamer SS2-55 can realize the high-throughput screening of OPs [71,72]. Zhang et al. [73] constructed an aptamer SS2-55-modified micro-interdigitated electrode chip for quantitative detection of the four OPs. The LODs for the target OPs were 0.24–1.67 fM. Sensitive detection was also realized in the SERE detection. An aptamer SS2-55-modified SERS platform was used to detect the four OPs with LODs of 0.4–24 mM [74]. By using broad-spectrum aptamers, the screening efficiency can be remarkably improved.

#### 2.2.3. Preparation and Application of Truncated Aptamers with Broad Specificity

Broadly specific aptamers can also be obtained by aptamer truncation besides selection strategy. The nucleotides contributing to target binding are designed and reserved. Therefore, broadly specific aptamers obtained by truncation usually contain relatively few bases. For example, a shortened 8-mer broadly specific aptamer with high affinity to four different TCs has been truncated from an original 76-mer aptamer [75]. The 8-mer aptamer conserved only the original binding pocket, conferring the stacking interaction with the same binding mechanism for the tested TCs. Then, the truncated aptamer was used in an AuNPs colorimetric assay. The sensitivity of the detection method for TCs was improved 500-fold, compared to that of the assay using the 76-mer aptamer.

Truncated broadly specific aptamers can be flexibly applied in the design of aptasensors. Zhou et al. [76] utilized the 8-mer aptamer to design a fluorescent aptasensing approach based on catalytic hairpin assembly and displacement of the G-quadruplex DNA. The sensitive detection of the four TCs in milk was achieved, with an LOD of 4.6 μg/L. Furthermore, a novel colorimetric triple-helix molecular switch aptasensor has also been designed using the same aptamer [77]. The assay showed selectivity toward the TCs with an LOD of 266 pM. Due to the short length of truncated broadly specific aptamers, various DNA-based amplification strategies can be realized. Meanwhile, the cost of aptamer synthesis can also be reduced.

#### 2.2.4. Preparation and Application of Multi-Aptamers

Aptamers can be easily synthesized and obtained after the sequences of the selected aptamers have been released in the literatures. Therefore, multi-analyte analyses of residues with multi-aptamers have attracted significant interests, with respect to constructing rapid and high-throughput screening methods. Multiple-label strategies and multiple-channel strategies are commonly used in multi-analyte analyses.

By using a multiple-label strategy, multi-analyte analysis can be realized within a single system. Different fluorophores [78,79], metal nanoparticles [80], and lengths of complementary DNA strands [81] have been used in the aptasensors for the simultaneous detection of multiple analytes. A multiplex aptasensor based on a fluorescence resonance energy transfer strategy has been developed for the triple-target detection of antibiotics [79]. Three aptamers specific to sulfadimethoxine, KAN, and ampicillin were labeled with cyanine 3, 6-carboxyfluorescein (FAM), and cyanine 5, which can be quenched by graphene oxide. Combined with a DNase I-assisted cyclic enzymatic signal amplification method, the sensitivity was improved with LODs of 1.997–2.664 ng/mL for the three antibiotics. Aside from fluorometric analysis, a dual-target metal ions based electrochemical aptasensor was developed for the simultaneous detection of KAN and streptomycin [80]. Metal ions (Cd^2+^ and Pb^2+^) which could generate distinct differential pulse voltammetry peaks were used as signal tracers. The LODs for KAN and streptomycin were 74.50 and 36.45 pM, respectively. In addition to being labeled with different signal traces, novel magnetic tripartite DNA assembly nanostructure probes have been constructed for the detection of KAN, aflatoxin M1, and 17β-estradiol [81]. Combined with a rolling circle amplification, three different lengths of complementary DNA strands were produced, which could be quantitated by the microfluidic chip. The LODs for KAN, aflatoxin M1, and 17β-estradiol were 0.32, 0.95, and 6.8 pg/mL, respectively.

**Figure 5:**
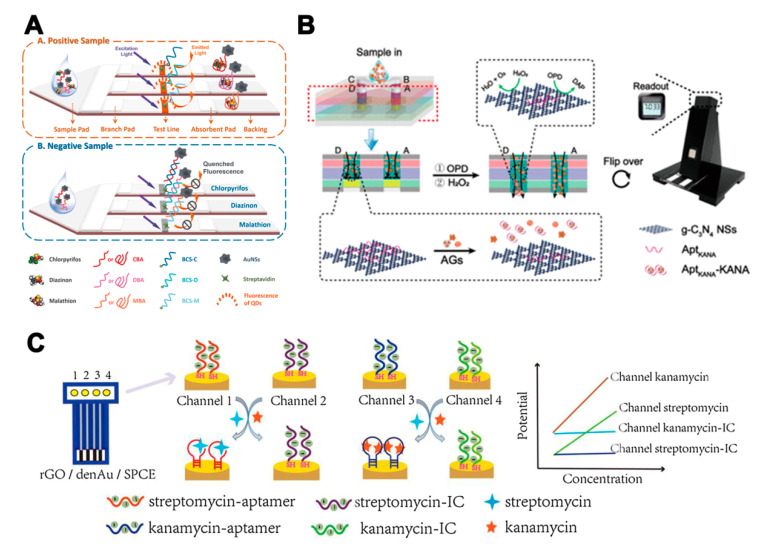
The assays based on multi-aptamers in multiple-channel strategies. (**A**) Illustration of a fluorescent aptamer-based lateral flow biosensor consisting of three specific test lines for the detection of three pesticide residues [82]. (**B**) Illustration of a work mode for the paper-based device digital fluorescence detector platform. The red dotted line shows the location of the cross-section. The left channel B–D is the assay region and the right channel A is the control region [83]. (**C**) Illustration of a novel potentiometric aptasensor array for the simultaneous detection of streptomycin and kanamycin with dual-channel internal calibration [84]. Reproduced with permission from [82]. Copyright Elsevier, 2018. Reproduced with permission from [83]. Copyright American Chemical Society, 2019. Reproduced with permission from [84]. Copyright Elsevier, 2020.

When a higher number of target analytes needs to be detected, multiple-channel strategies, which feature multiplex channels or reaction positions, present advantages. Cheng et al. [82] developed a fluorescent aptamer-based lateral flow biosensor consisting of three specific test lines, in order to detect three pesticide residues. A novel fluorophore-quencher nano-pair (QDs and gold nanostars) was implemented to label the aptamers for signal amplification (Figure 5A). The LODs of chlorpyrifos, diazinon, and malathion were 0.73, 6.7, and 0.74 ng/mL, respectively. In addition to conventional lateral flow assays, a novel quantitative reaction platform with four parallel channels has been used for the detection of three aminoglycosides [83]. For each channel, a specific aptamer and graphitic carbon nitride nanosheets complex catalyzed o-phenylenediamine to fluorescent 2,3-diaminophenazine. When the target was added, the aptamer was removed from nanosheets, leading to a decrease of the fluorescent signal (Figure 5B). Sensitivity analyses of streptomycin, tobramycin, and KAN were realized simultaneously. Moreover, multiple-channel strategies can also be used in the development of electrochemical aptasensor. A novel 4-channel potentiometric aptasensor array was developed for the detection of streptomycin and KAN [84]. A dual-internal calibration system was used to avoid the influence of the background matrix (Figure 5C). The aptasensor demonstrated high sensitivity for the detection of streptomycin and KAN with LODs of 9.66 and 5.24 pM, respectively.

### 2.3. Rapid Multi-Residue Detection Methods Based on Molecularly Imprinted Polymers

MIPs have been identified as another substitute for antibodies, due to their advantages of low cost, high chemical stabilities, and specific recognition [85]. The conventional procedures for MIP fabrication involve the polymerization of templates and functional monomers in the presence of cross-linkers. After the template is removed from the 3D polymer network, specific recognition cavities were remained, which have complementary functionality and shape to the template [86]. The molecular recognition mechanism is mainly based on the intermolecular interactions between the template and functional groups in the polymers [87]. MIPs have been applied to analyte separation and sensor analysis [88]. Due to the favorable stabilities and low binding capacities, MIPs are usually used to isolate multiplex target analytes from complex food matrixes [89]. For multi-residue detection, MIPs have been applied as recognition elements with good anti-matrix effects. However, MIPs fabricated by traditional methods have high selectivity to the target analyte, which limits their application in multi-analyte analysis. The molecule that shares a common structure and functional groups with target analytes can be used as a template for the synthesis of broadly specific MIPs.

Broadly specific MIPs have been used as recognition elements in biomimetic ELISA, electrochemical assays, and SERS assays for multi-residue detection. MIPs were used as an antibody substitute to develop a biomimetic ELISA method for the detection of trichlorfon and acephate. A 4-(Dimethoxyphosphorothioylamino) butanoic acid sharing a common structure with OPs pesticides was used as a template molecule [90]. The LODs for trichlorfon and acephate were 8.0 and 12.0 μg/L, respectively. These MIPs were also used to develop a novel biomimetic electrochemical sensor for the detection of trichlorfon and acephate [91]. The LODs were 8.94 × 10^−12^ and 6.81 × 10^−11^ M for trichlorfon and acephate, respectively. Furthermore, another molecule sharing a common structure with OP pesticides, diethylphosphonoacetic acid, has been used as a template to synthesize MIPs [92]. The obtained MIPs were used to develop an electrochemical sensor for the quantification of methamidophos and omethoate. This method was ultrasensitive for the detection of methamidophos and omethoate, with LODs of 2.67 × 10^−13^ and 2.05 × 10^−14^ mol/L, respectively. Furthermore, surface plasmon resonance sensor fabricated molecular imprinting nanofilms were developed for the detection of three pesticides [93]. The LODs of cyanazine, simazine, and atrazine were 0.095, 0.031, and 0.091 nM, respectively. Therefore, multi-residue detection based on MIPs has several advantages, such as short response time and good signal stability, which provide great potential for the detection of pesticides and veterinary drugs.

## 3. Rapid Multi-Residue Detection Methods Based on the Inherent Characteristics of Pesticides and Veterinary Drugs

Aside from the above mentioned multi-residue detection methods with different recognition elements, enzymatic inhibition-based sensors, NIR spectroscopy, and SERS spectroscopy have also been used for the multi-analyte analysis of pesticide and veterinary drug residues, with the development of bio-neuroinformatics technology, spectroscopy technology, and nanotechnology. These detection methods were mainly developed based on the inherent characteristics of pesticides and veterinary drugs. Therefore, these methods can realize non-destructive, rapid, low cost, and environmentally friendly detection without the use of recognition elements. Enzymatic inhibition-based biosensors have been successfully applied in multi-residue screening and detection, based on the enzyme inhibition characteristics of pesticide residues. Due to the specific functional groups and chemical structures of pesticides and veterinary drugs, different absorption bands and spectral intensities are shown in the NIR or SERS spectrum. Thus, enzyme-based sensors, NIR spectroscopy, and SERS spectroscopy have become promising detection techniques for the multi-analyte analysis of residues.

### 3.1. Enzymatic Inhibition-Based Multi-Residue Detection

The activities of cholinesterases, acetylcholinesterase(AChE), and butyrylcholinesterase can be inhibited by OP and carbamate (CM) pesticides [94]. Based on this principle, enzymatic inhibition-based multi-residue detection can be applied in multi-analyte monitoring [95]. Quantitative detection can also be achieved, as the degree of inhibition is related to the concentration of pesticide residues [94]. The phosphorus atoms of the OPs can covalently bind to the hydroxyl group of the nucleophilic serine located at the active site of AChE [94]. Therefore, AChE is the most commonly used enzyme in the development of enzymatic inhibition-based multi-residue detection methods. Various detection methods based on enzymatic inhibition have been published, including colorimetric assays and electrochemical assays.

AChE can hydrolyze certain substrates to colored products, while the color development can be decreased in the presence of OP or CM pesticides [96]. Therefore, various colorimetric screening methods have been developed based on this principle. An AChE assay has been optimized and validated for carbofuran, carbofuran-3-hydroxy, and dichlorvos in lettuce and strawberry extracts [97]. Indoxyl acetate was used as a substrate which can be rapidly hydrolyzed by AChE to produce blue hydrazine. The LODs of three kinds of OP and CM pesticides were found to be at the part per billion (ppb) level. Furthermore, this reaction was integrated into a double-film visual screening card for the rapid screening of OP or CM pesticides [98]. The LODs of six OP and CM pesticides were in the range of 0.04–0.5 mg/mL. With the development of nanotechnology, diverse nanomaterials with excellent enzyme immobilization properties have been used to develop electrochemical assays. An AChE electrochemical biosensor based on gold nanorods (AuNRs) was developed for the detection of OP pesticides. The LODs of paraoxon and dimethoate were 0.7 and 3.9 nM, respectively [99]. Graphene and transition metal carbides have also been used to modify an AChE biosensor for the detection of OP pesticides. The biosensor showed better catalytic performance compared to the biosensors without modification [97]. Furthermore, a robust and novel conjugated polymer and core-shell magnetic nanoparticle containing biosensor was applied to detect pesticides [100]. The biosensor revealed a rapid response of 5 s and an LOD of 6.66 × 10^−3^ mM.

The above detection methods can be used for the rapid pre-screening of OP or CM pesticides, in order to discriminate whether the sample contains pesticides or not. No qualitative or quantitative information about individual pesticides in the complex matrix can be obtained. Therefore, multi-sensor arrays combined with artificial neural networks (ANNs) have been developed for multi-residues quantitative analysis. Different types of native or genetically engineered AChEs portrayed different sensitivities to OP or CM pesticides, which were used to modify the multi-biosensors. Combined with the data processing of ANNs, the pesticide mixtures could be discriminated [95]. An AChE triple-biosensor array combined with an ANN was developed for the selective detection of chlorpyrifos and chlorfenvinfos [101]. Three different types of AChE, the wild-type from an electric eel, the genetically modified *Drosophila melanogaster* AChE B394 and B394, were used to modify the biosensors. The combined responses of the two pesticides were modeled by two different ANNs. Both pesticides could be quantified with low errors from a direct measurement step. Moreover, one-step AChE disposable biosensors based on genetically modified enzymes and ANNs has been developed to determine mixtures of three OP residues [102]. Two AChEs from *Drosophila melanogaster* (wild-type and genetically modified) were used to modify the biosensor array. The biosensor system successfully identified and quantified mixtures of chlorpyriphos oxon, chlorfenvinphos, and azinphosmethyl oxon.

Enzymatic inhibition based multi-residue detection methods have been applied in the rapid pre-screening of OP and CM pesticides. However, there are still some problems which need to be solved. One is how to retain the original catalytic activity of AChE after immobilization. Therefore, the immobilization surface should be well-designed. The second is the issue of the re-activation of an enzymatic layer complicating the operation of a biosensor. Therefore, a disposable biosensor with cheap production cost is desirable for field conditions. The third is a selectivity problem. It is difficult to determine which kinds of OPs inhibit the AChE activity in a particular sample. Although multi-sensor arrays combined with ANNs can provide a partial solution this problem. Due to the needs of different AChEs and the complex processing of ANNs, the practicality and operability need to be further improved.

### 3.2. Near-Infrared Spectroscopy Based Multi-Residue Detections

NIR spectroscopy methods have been recognized as a non-destructive evaluation approach for the quality control of intact food [103]. Aside from quality assessment and food compounds measurement, NIR spectroscopy has been shown to have feasibility for the detection of pesticide and veterinary drug residues, in order to ensure food safety [104].

Typically, NIR absorption spectra are collected and the spectral information is analyzed for the assessment of residues. Jamshidi et al. [105] applied visible/near-infrared (Vis/NIR) spectroscopy in the range of 450–1000 nm to detect pesticide residues in intact cucumbers. Partial least squares-discriminant analysis (PLS-DA) models were developed to classify cucumbers with contents of diazinon below and above the maximum residue limits as safe and unsafe samples, respectively. Furthermore, Vis/NIR spectroscopy combined with a chemometric method has been developed for the detection of pesticide residues in cucumber samples [106]. Partial least squares (PLS) regression models and PLS-DA models were developed for the prediction of diazinon contents in the samples. For the convenience of users, a graphical user interface was created, based on the PLS and PLS-DA models, for the classification of cucumbers by the absence/presence of diazinon residues, respectively.

However, the spectra used in the above methods were collected from a small portion of the tested samples, which cannot guarantee the data accuracy and representativeness. Therefore, hyperspectral imaging technology has been used, in combination with NIR spectroscopy methods. Hyperspectral imaging integrated spectroscopy and imaging can be used to obtain both spectral and spatial information from samples [107]. An NIR-hyperspectral imaging technique and GC-MS were used to detect two pesticides (chlorpyrifos and imidacloprid) in jujube fruits in the spectral 900–1700 nm. Based on the extracted spectral data, a simplified E_S_-AWLSGSD-RC-LWPLSR model was established with eight characteristic wavelengths, having correlation coefficients of cross-validation of 0.757 and 0.898 for chlorpyrifos and imidacloprid, respectively [108]. Moreover, an NIR-hyperspectral imaging system has also been used to map the distribution of pesticide residues in mulberry leaves [107]. The best successive projections algorithm multiple linear regression model was used to transfer the distribution of the pesticide residues to a visualization map. Furthermore, an NIR-hyperspectral imaging system (870–1780 nm) combined with a chemical molecular structure coupled with wavelet transform was proposed to detect five kinds of pesticide residues (dimethoate, acephate, phoxim, dichlorvos, and avermectin) on the surface of lettuce [109]. The calibration and prediction accuracy of the results were achieved at 100%.

NIR spectroscopy methods are capable of realizing multi-residue detection in intact food. In addition to the characteristic of non-destructive, NIR spectroscopy has the advantages of no sample preparation needed, fast response time, environmental friendliness, and low operating cost. Therefore, NIR spectroscopy methods have a great application potential in online monitoring and field detection.

### 3.3. Surface-Enhanced Raman Scattering Based Multi-Residue Detections

SERS is a promising method, combining Raman spectroscopy and nanotechnology for the rapid and non-invasive detection of samples. The chemical bonds and vibrational properties of functional groups are represented by Raman spectral bands, providing a fingerprint for target analytes [10]. With the enhancement effect of SERS substrates, SERS can identify and quantify analytes with high sensitivity down to the single-molecule level. Various SERS substrates have been designed to functionalize the surface characteristics, in order to enhance the sensitivity of SERS [110]. Improved SERS substrates provided the possibility of multi-residue detection within a complicated matrix [111].

**Figure 6:**
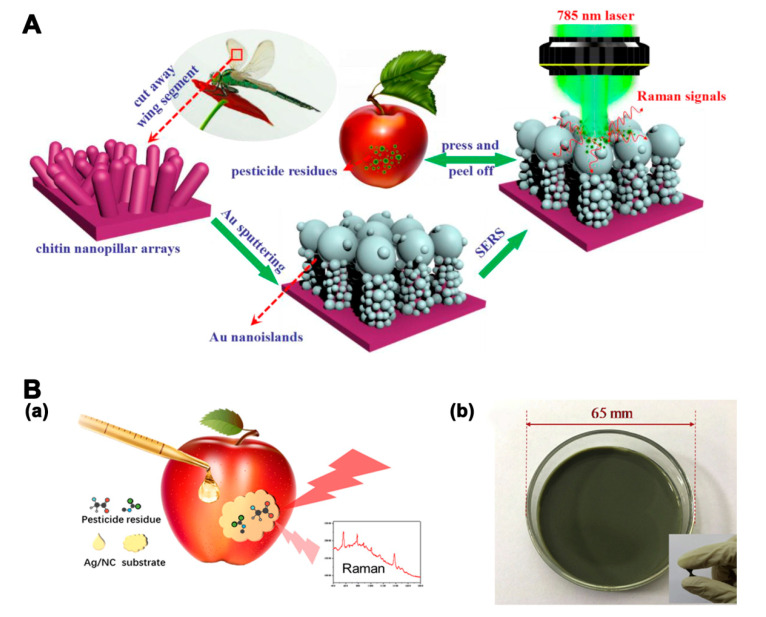
(**A**) Illustration of an au-decorated dragonfly wing bioscaffold array as a surface-enhanced Raman scattering (SERS) substrate by the Raman system [115]. (**B**) a. Illustration of the SERS measurement of apples. b. Photograph of the Ag/NC substrate [117]. Reproduced with permission from [115]. Copyright MDPI, 2018. Reproduced with permission from [117]. Copyright Elsevier, 2019.

The sensitivity of SERS is influenced by the molecular intrinsic vibration of the targets and the interaction between targets and the SERS substrate. Pesticides and veterinary drugs with certain thiol or amine functional groups can bind strongly to Au and Ag substrates [112]. Therefore, various Au and Ag SERS substrates have been applied for the simultaneous detection of pesticide and veterinary drug residues. An in situ SERS method with 50 nm AuNPs as substrates was developed to discriminate three classes of pesticides on fresh tea leaves and apple peels [113]. In addition to single materials, silver-coated gold nanoparticles (Au@Ag NPs) have also been used as a substrate for the rapid detection of multiple OPs [114]. The Raman enhancement of composited Au@Ag NPs for OPs detection was stronger than that of single Ag and Au NPs. The aggregation of NPs caused by the connection between Au@Ag NPs and analytes could create hot spots, which showed significant Raman enhancement. The LODs of triazophos and methyl-parathion in peach were 0.001 mg/kg. For practical applications, flexible SERS substrates consisting of SERS active nanomaterials and flexible solid substrates are useful for the detection of surface residues. Wang et al. [115] constructed an Au-decorated dragonfly wing bioscaffold array as a flexible SERS substrate for the detection of three kinds of pesticides. The SERS substrate, fabricated by a 3D biomimetic array with high-density hot spots, showed greatly enhanced Raman activity (Figure 6A). The SERS platform was used for the detection of acephate, cypermethrin, and tsumacide, with respective LODs of 10^−3^, 10^−3^, and 10^−4^ ng/cm^2^. A nanoporous cellulose paper-based AuNRs SERS substrate was constructed for the detection of three pesticides [116]. The LODs of thiram, tricyclazole, and carbaryl were 6, 60, and 600 ng/cm^−2^, respectively. Chen et al. [117] developed a jelly-like flexible SERS substrate based on nanocellulose decorated with Ag nanoparticles (Ag/NC substrate) for the detection of two types of pesticides on apple peels and cabbages (Figure 6B). The flexible SERS substrate facilitated good contact with the samples. The LODs for thiram and thiabendazole were 0.5 and 5 ng/cm^2^, respectively.

SERS has demonstrated excellent sensitivity, simple sampling, and rapid test speed for the multi-residues detection of pesticide and veterinary drug residues. With facile design, SERS substrates with the advantages of flexibility, excellent adaptability, cost-efficiency, and suitability for large-scale production can be constructed for non-destructive practical applications. With the development of nanotechnology and the improvement of instruments, SERS can provide a routine and cost-effective detection method for the multi-analyte analysis of pesticide and veterinary drug residues.

## 4. Conclusions

The residues of pesticides and veterinary drugs threaten human health. Therefore, several screening assays have been developed for the rapid multi-residue detections of pesticides and veterinary drugs. Different broadly specific recognition elements have been prepared and applied for simultaneous analysis. In addition, enzymatic inhibition-based sensors, NIR spectroscopy, and SERS spectroscopy, based on the inherent characteristics of pesticides and veterinary drugs, can also realize multi-analyte analysis. With the development of genetic engineering and computer modeling technology, recognition elements with the desired broad specificity and high affinity to multiplex targets can be designed and obtained. Meanwhile, the design of novel portable instruments and the synthesis of novel functional materials serve to promote the development of new detection methods with more target analytes and higher sensitivities. With the combination of rapid multi-analyte analysis methods and traditional instruments, efficient and accurate detection systems which feature simplified test processes and reduced determination time can be built.
